# Metaviromic Profiling of Genetic Diversity and Prevalence of Suid Herpesviruses Circulating in China

**DOI:** 10.3390/v18060625

**Published:** 2026-05-29

**Authors:** Jing Wang, Zhibin Shi, Shida Wang, Zaisi Liu, Lili Wei, Jingfei Wang

**Affiliations:** 1 College of Veterinary Medicine, Northeast Agricultural University, Harbin 150030, China; 2 State Key Laboratory of Animal Disease Control and Prevention, Harbin Veterinary Research Institute, Chinese Academy of Agricultural Sciences, Harbin 150069, China; 3 Observation and Research Data Center for Animal Health, Ministry of Agriculture and Rural Affairs, Harbin 150069, China

**Keywords:** suid herpesviruses, nasal swab, serum, PoCMV, metavirome analysis

## Abstract

Three species of suid herpesviruses (SuHVs) have been reported in pigs, specifically pseudorabies virus (PRV), porcine cytomegalovirus (PoCMV), and porcine lymphotropic herpesvirus (PLHV). However, their genetic diversity and epidemic circulating status in China remain largely unclear. In this study, 7200 nasal swabs and 2571 serum samples were collected from pigs across 17 provincial regions in China in 2017. All samples were pooled into 22 libraries based on sample type and geographic origin for high-throughput next-generation sequencing. Metaviromic analysis identified all three SuHV species, revealing marked variations in their detection rates and viral abundance. Notably, PoCMV and PRV were detected in all 17 sampled provinces, accompanied by high viral genome sequence abundance (RPM > 1 × 10^2^), while PLHV was only found in nasal swabs from 10 provinces, with extremely low sequence abundance (RPM < 2). Further phylogenetic and genetic diversity analyses revealed notable molecular characteristics of the three circulating SuHVs: PoCMV exhibited substantial genetic diversity with at least two major evolutionary clades identified in Chinese pig populations; variant genotype II PRV strains were confirmed as the predominant circulating lineage; and potential PLHV variants with partial sequence divergence from the reference strain were also found to circulate in China. These findings enrich the molecular epidemiological data of SuHVs in Chinese pig populations and highlight the previously overlooked, highly widespread circulation of PoCMV, warranting attention to its potential impacts on swine health and production performance.

## 1. Introduction

Suid herpesviruses (SuHVs) are a group of double-stranded DNA viruses belonging to the family *Herpesviridae* and represent major viral pathogens threatening the global swine industry [1]. Three distinct SuHV species have been well characterized in domestic pigs, namely, pseudorabies virus (PRV, SuHV-1), porcine cytomegalovirus (PoCMV, SuHV-2), and porcine lymphotropic herpesvirus (PLHV, SuHV-3) [2]. Among them, PRV has long been recognized as a highly contagious, neurotropic, and zoonotic pathogen, causing severe reproductive disorders in sows and lethal encephalitis, respiratory distress, and high mortality in neonatal pigs; it is also capable of cross-species transmission to ruminants, companion animals, and even humans, leading to life-threatening clinical complications such as encephalitis and endophthalmitis in humans [3,4].

In contrast to the extensive attention paid to PRV, PoCMV and PLHV have long been overlooked in routine swine health monitoring and epidemiological investigations. PoCMV, a betaherpesvirus, commonly causes subclinical persistent infection in pigs, but can induce systemic disease, reproductive failure, and growth retardation in neonatal and young pigs under immune stress, potentially causing production losses and facilitating co-infection with other swine pathogens [5,6]. PLHV is a lymphotropic gammaherpesvirus that infects porcine lymphocytes and may be associated with immunosuppression and lymphoproliferative disorders in pigs [7,8]. However, its pathogenicity and epidemiological distribution remain poorly defined.

Despite the acknowledged threat posed by SuHVs, comprehensive molecular epidemiological data regarding their detection patterns, genetic diversity, geographic distribution, and viral abundance in Chinese pig populations remain limited, particularly for the historically neglected PoCMV and PLHV. Traditional diagnostic methods, including serological testing and conventional PCR, often fail to achieve unbiased, high-throughput detection of multiple viral pathogens simultaneously, thereby hindering a thorough understanding of SuHV epidemiology on a nationwide scale [9,10,11,12]. Metaviromic sequencing based on next-generation sequencing (NGS) technology enables unbiased identification and quantitative analysis of viral communities in clinical samples, providing a powerful tool to reveal the prevalence, genetic characteristics, and circulation patterns of both dominant and understudied viruses [13,14,15].

To address these research gaps, the present study collected a large panel of clinical samples from pigs across 17 provincial regions in China and performed metaviromic sequencing to systematically investigate the infection status, detection rates, viral abundance, and genetic diversity of three SuHV species. This study aims to enrich the molecular epidemiological database of SuHVs in China, highlight the widespread and previously overlooked circulation of PoCMV, and provide critical data for the prevention, control, and targeted surveillance of SuHV-associated diseases in the global swine industry.

## 2. Materials and Methods

### 2.1. Ethics Statement

The surveillance program and sampling strategy used in this study were reviewed and approved by the Animal Husbandry and Veterinary Bureau, Ministry of Agriculture and Rural Affairs, People’s Republic of China.

### 2.2. Sample Collection

**Figure 1:**
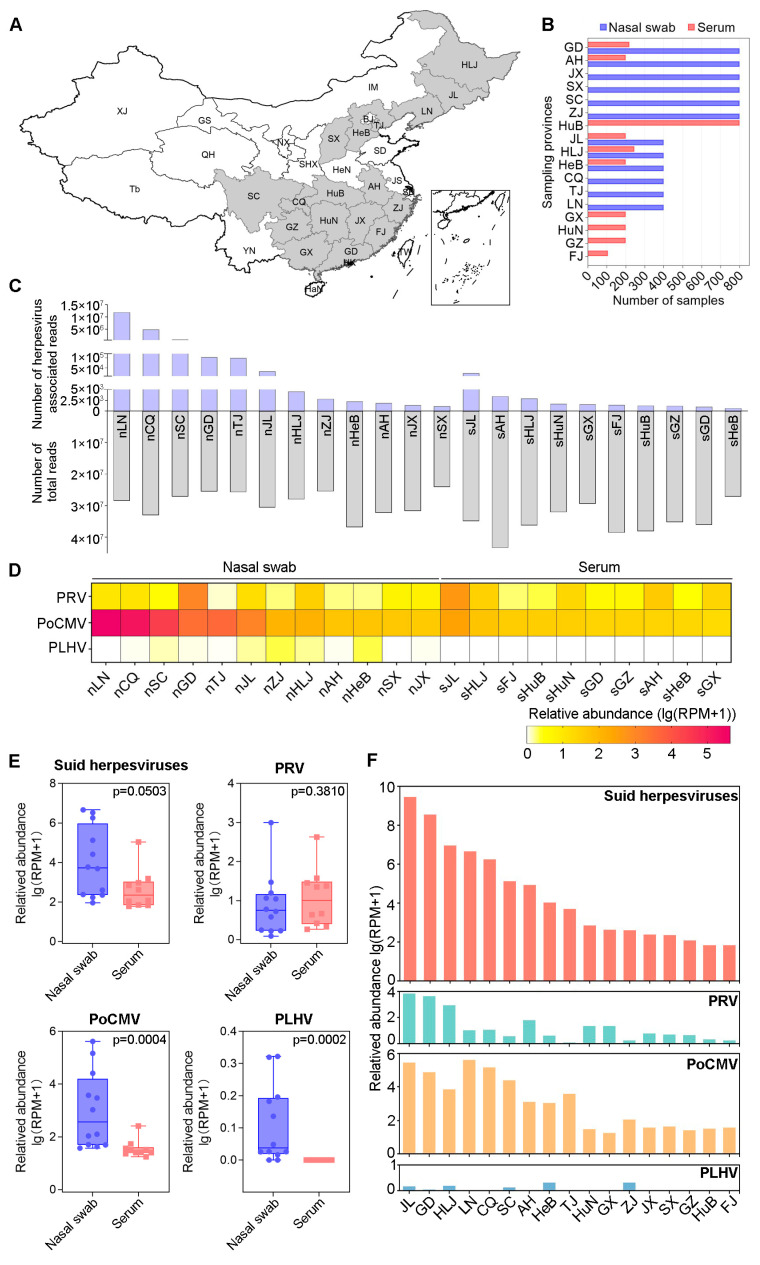
Investigation regions, sample numbers, and SuHV gene abundance. (**A**) Sampling regions and sample sizes. Sampling regions are colored gray. Abbreviations: HLJ, Heilongjiang; JL, Jilin; LN, Liaoning; BJ, Beijing; TJ, Tianjin; IM, Inner Mongolia; HeB, Hebei; SD, Shandong; SX, Shanxi; HeN, Henan; JS, Jiangsu; AH, Anhui; SH, Shanghai; ZJ, Zhejiang; FJ, Fujian; JX, Jiangxi; GD, Guangdong; GX, Guangxi; HuB, Hubei; HuN, Hunan; GZ, Guizhou; SC, Sichuan; CQ, Chongqing; SHX, Shaanxi; NX, Ningxia; GS, Gansu; QH, Qinghai; XJ, Xinjiang; Tb, Tibet; YN, Yunnan; HaN, Hainan; TW, Taiwan; HK, Hong Kong; Mc, Macao. The map was generated using QGIS software (v3.0). (**B**) Overview of the sample pooling strategy and library sizes across different provinces. The bar chart shows the total number of samples in each pooled library, stratified by sample type: blue bars represent nasal swab samples and red bars represent serum samples. (**C**) Sequencing depth and herpesvirus-associated read statistics for each pooled library. Gray bars represent the total number of raw reads obtained from each library and light purple bars represent the number of reads taxonomically classified as suid herpesvirus-associated. (**D**) Heatmap showing the abundance of SuHVs across 22 sequencing libraries. Abundance values range from 0 to 1 × 10^6^ RPM. “n” denotes nasal swab samples; “s” denotes serum samples. (**E**) Alpha-diversity analyses of SuHVs between the two sample types. Each box represents the median, upper quartile, and lower quartile of the estimates. The corresponding *p*-value is indicated above the box. (**F**) Relative abundance of SuHVs among different geographical regions.

A total of 9771 samples, including 7200 nasal swabs and 2571 serum specimens, were collected from clinically healthy pigs at slaughterhouses across 17 provincial regions in 2017 (Figure 1A). Each nasal swab was placed into 1.5 mL of sterile phosphate-buffered saline (PBS), while individual serum samples were stored in 1.5 mL microcentrifuge tubes. All samples were kept frozen at −20 °C until further processing.

### 2.3. Library Construction and Sequencing

All samples were grouped into 22 libraries based on sample type and geographic origin for metaviromic profiling (Figure 1B). Sample pretreatment and viral nucleic acid library construction were performed following previously established protocols with minor modifications [16,17]. Briefly, swab supernatants and serum were centrifuged at 13,000× *g* for 20 min to remove impurities, and the clarified supernatants were then sequentially filtered through 0.45 μm and 0.22 μm filters, followed by ultracentrifugation at 160,000× *g*, 4 °C for 4 h to concentrate viral particles. The resulting pellets were resuspended in PBS overnight and thoroughly mixed prior to enzymatic treatment. Extracellular host nucleic acids were eliminated by digestion with DNase I and RNase I. Viral DNA/RNA was extracted using the EasyPure Viral DNA/RNA kit (TransGen, Beijing, China). Random-primed reverse transcription and amplification were conducted as follows: the first-strand cDNA was synthesized using the K9N random primer (5′-GACCATCTAGCGACCTCCCANNNNNNNNN-3′) and PrimeScript II RTase (Takara, Dalian, China) at 42 °C for 3 h, followed by inactivation at 95 °C for 5 min; the second-strand cDNA was generated using DNA Polymerase I Large (Klenow) Fragment (Promega, Madison, WI, USA) at 37 °C for 3 h and then inactivated at 75 °C for 10 min. The resulting DNA/cDNA was amplified in 50 μL reactions containing 2× KOD FX Neo buffer, 0.5 mmol/L dNTP mixture, 5 μL nucleotide, 10 mmol/L K9 primer (5′-GACCATCTAGCGACCTCCCA-3′), and 1U KOD FX Neo DNA polymerase (Toyobo, Osaka, Japan). Thermal cycling conditions consisted of initial denaturation at 94 °C for 2 min, followed by 40 cycles at 98 °C for 10 s, 55 °C for 30 s, and 68 °C for 2 min. Amplification products were verified using 1% agarose gel electrophoresis. A total of 6 μg of purified PCR products of 22 sample libraries were submitted to Shanghai Personalbio Technology Co., Ltd., Shanghai, China, for paired-end sequencing (2 × 150 bp) using the Illumina HiSeq 2500 platform (San Diego, CA, USA).

### 2.4. Herpesvirus-Associated Viral Genome Discovery

Raw sequencing reads were subjected to quality control and trimming using the BBTools software package (v39.81) (https://archive.jgi.doe.gov/data-and-tools/software-tools/bbtools/, accessed on 26 May 2026). Reads corresponding to ribosomal RNA were removed by mapping against a comprehensive rRNA database retrieved from the SILVA database 138.2 (https://www.arb-silva.de/, accessed on 26 May 2026) using Bowtie2 software (v1.2.2) [18]. Clean reads were aligned to a custom local database composed of full-length genomic sequences of the family *Herpesviridae*, with an E-value threshold of 1 × 10^−4^. Viral abundance was quantified as reads per million (RPM). To obtain longer viral contigs, rRNA-free reads were subjected to de novo assembly using the MEGAHIT program (v1.2.6) [19]. Assembled contigs were further validated and extended by remapping reads to reference viral genomes using Bowtie 2 (v2.3.5.1) again.

### 2.5. Phylogenetic Analysis

All sequences were aligned using the ClustalW algorithm implemented in Geneious Prime (v 2025) [20]. Maximum likelihood (ML) phylogenetic trees were constructed using MEGA (v 6.06), employing 1000 bootstrap replicates to assess topological confidence [21]. Similarity analysis of the full-length PoCMV genome was conducted using SimPlot software (v 3.5.1) [22].

### 2.6. Statistical Analysis

All statistical analyses and visualizations were performed using R software (v 4.5.2). Viral abundance values (RPM) were normalized prior to comparative analysis. A heatmap displaying the abundance distribution of the three SuHV species across libraries was generated using the pheatmap package (v1.0.13) with default settings. Viral alpha diversity was analyzed to compare community structure between the nasal swab and serum groups. Differences in alpha diversity indices were evaluated using a two-tailed, unpaired, nonparametric Mann–Whitney U test. A *p*-value < 0.05 was considered statistically significant.

### 2.7. Nucleotide Sequence Accession Number

The complete sequences of the *gB*, *MCP*, and *DPOL* genes reported in this paper have been deposited in the GenBase [23] of the National Genomics Data Center [24], Beijing Institute of Genomics, Chinese Academy of Sciences/China National Center for Bioinformation. These sequences are available under accession numbers C_AA167569.1 to C_AA167578.1 and can be accessed publicly at https://ngdc.cncb.ac.cn/genbase.

## 3. Results

### 3.1. Diversity and Detection Patterns Characteristic of SuHVs

To survey the genetic diversity and detection patterns of SuHVs in pigs in China, we conducted metaviromic analyses on 22 libraries (12 nasal swabs, 10 sera), representing more than 9771 pigs from 17 provinces in China (Figure 1A,B). Following sequencing, the reads contained in each library ranged from 24,045,847 (nSX) to 43,245,512 (sAH). Among them, 648 (sHeB) to 11,887,863 (nLN) reads were classified as belonging to the *Herpesviridae* family (Figure 1C). All three species of SuHV (PRV, PoCMV, and PLHV) were detected in this study. Notably, PRV and PoCMV exhibited high abundance across all libraries, with RPM values exceeding 1 × 10^2^ in each library. In contrast, PLHV was only identified in 10 libraries at extremely low abundance (Figure 1D). This large-scale metaviromic survey reveals the widespread circulation of SuHVs in Chinese pig populations. While PRV is a well-recognized swine pathogen, our data demonstrate that PoCMV is also highly abundant and geographically widespread. Conversely, PLHV showed limited detection and extremely low abundance. These findings underscore the urgent need to re-evaluate the epidemiological significance of PoCMV and incorporate it into future monitoring programs for swine herpesviruses in China.

To assess differences in SuHV abundance between the two sample types, the Shannon index was calculated, revealing no significant difference between nasal swabs and sera (*p* = 0.0503). We further compared the Shannon index for each viral species between sample types, showing that the index values for PoCMV (*p* = 0.0004) and PLHV (*p* = 0.0002) were significantly higher in nasal swabs than in sera, whereas no significant difference was observed for PRV (Figure 1E). These results indicate that the overall alpha diversity of SuHV is significantly higher in nasal swabs than in serum samples. Notably, the diversity of PoCMV and PLHV exhibits a similar tissue distribution pattern, with significantly higher diversity in nasal swabs, while PRV diversity was comparable between the two sample types. These findings suggest that nasal swabs may represent a more suitable sample type for evaluating the diversity and infection status of certain SuHV species, particularly PoCMV and PLHV.

To investigate the spatial distribution of SuHVs across the sampled provinces, we ranked the provinces according to the overall abundance of SuHVs and generated histograms for PRV, PoCMV, and PLHV, respectively (Figure 1F). Overall, SuHVs exhibited a high detection frequency in all sampled provinces, with a clear geographic gradient in relative abundance. PRV displayed a geographically restricted high-abundance pattern, maintaining elevated relative abundance in Jilin, Guangdong, and Heilongjiang, while its abundance was markedly lower in other provinces. PoCMV showed consistently high relative abundance across all sampled provinces, with no obvious geographic variation. PLHV was only sporadically detected in nasal swab libraries from a limited number of provinces, including Jilin, Heilongjiang, Sichuan, Hebei, and Zhejiang, and exhibited low relative abundance. These results demonstrate considerable variation in the abundance of SuHVs in Chinese pig populations, with each viral species presenting a distinct geographic distribution profile.

### 3.2. Phylogenetic Evolution of SuHVs

**Figure 2:**
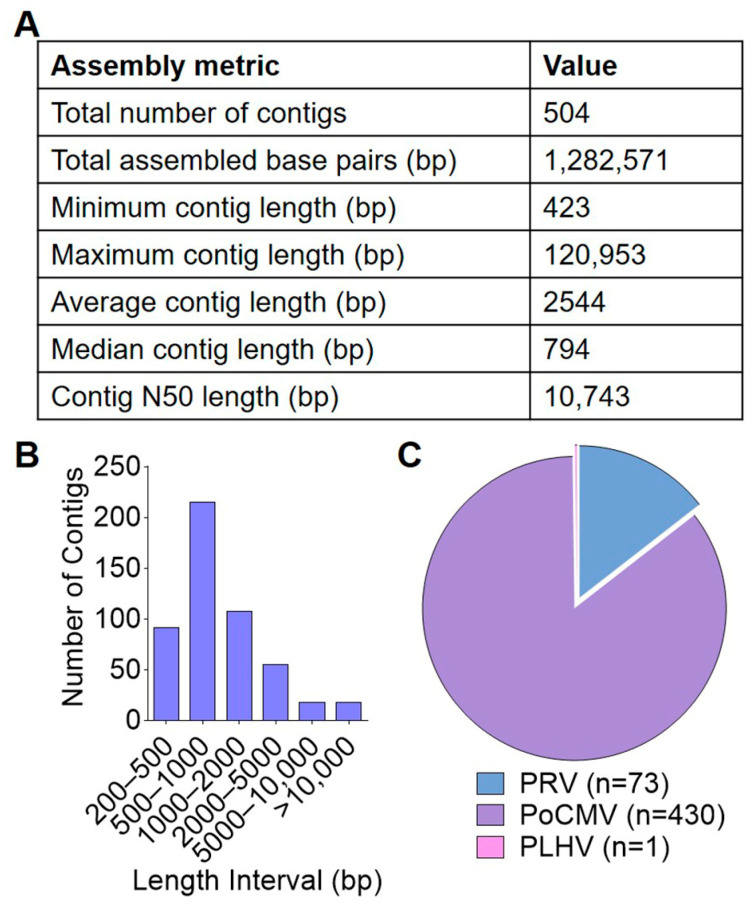
Assembly and classification of contigs derived from high-throughput sequencing. (**A**) Summary statistics of the de novo assembly. (**B**) Length distribution of all 504 assembled contigs. (**C**) Distribution of contigs assigned to different suid herpesviruses. The pie chart shows the proportion of contigs matching PRV, PoCMV, and PLHV.

To understand the genetic evolution of SuHVs identified in this study, we performed de novo assembly based on the obtained herpesvirus-associated reads. A total of 504 contigs were generated, with a cumulative length of 1,282,571 bp (Figure 2A). The contig lengths ranged from 423 bp to 120,953 bp, with an average length of 2544 bp. The N50 contig length reached 10,743 bp, indicating acceptable continuity and completeness of the assembly. Length distribution analysis showed that most contigs were concentrated in the 500~1000 bp (*n* = 214, 42.5%), followed by the 1000~2000 bp interval (*n* = 107, 21.2%). A small proportion of long contigs (>10,000 bp, *n* = 18, 3.6%) was also obtained, supporting further genomic characterization (Figure 2B). Viral classification analysis revealed that the majority of contigs (*n* = 430, 85.3%) were assigned to PoCMV, while 73 contigs (14.5%) matched PRV. Only one contig was identified as PLHV, indicating that PoCMV was the dominant viral species in the sample (Figure 2C).

**Figure 3:**
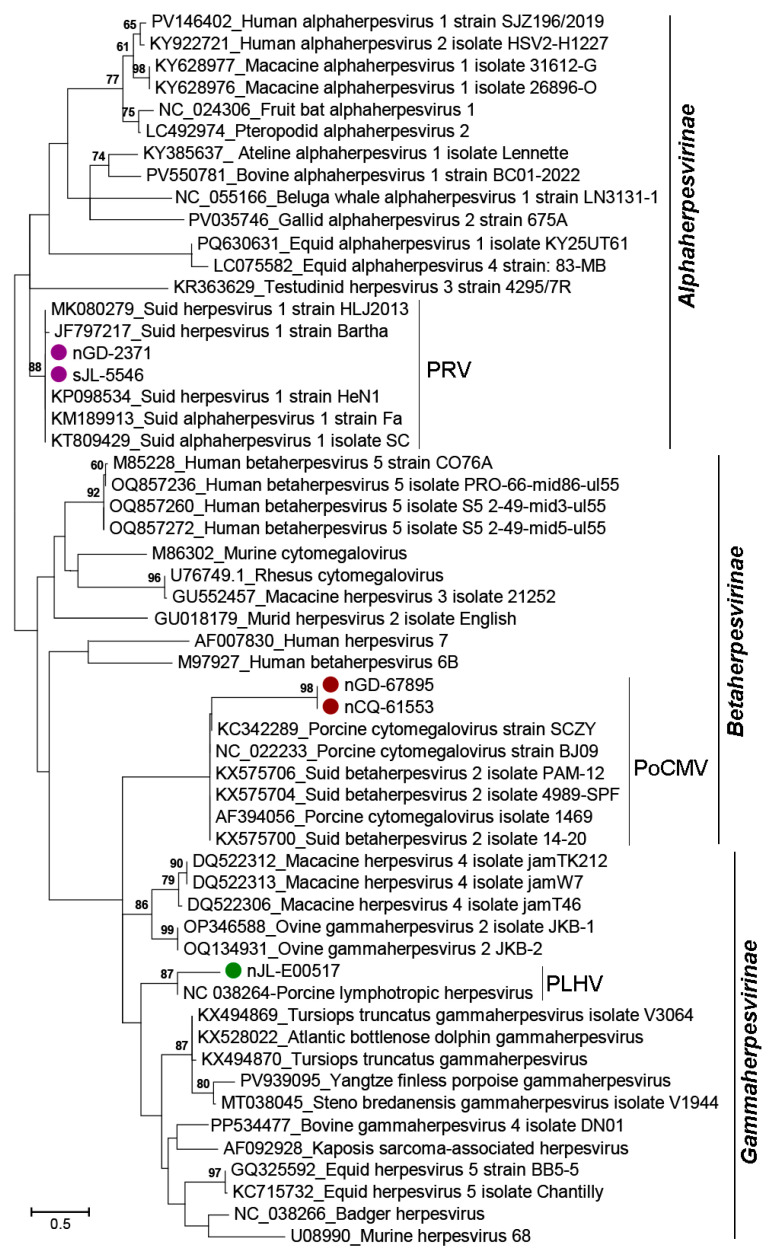
Maximum likelihood (ML) phylogenetic tree of SuHVs. The ML tree of viruses in the family *Herpesviridae* was constructed based on the partial *gB* sequence (positions 16,474–16,640, referenced to the genome sequence of PRV strain HLJ2013) using MEGA 6.06, with 1000 bootstrap replicates. PRV, PoCMV, and PLHV identified in the present study are indicated by purple, red, and green solid circles, respectively. The genera of SuHVs are annotated on the right side of the corresponding branches.

To further explore the phylogenetic characteristics of the SuHVs identified in this study, we constructed a maximum likelihood (ML) phylogenetic tree based on the partial *gB* gene sequences of SuHVs identified in this study and 61 reference strains, including 25 alphaherpesviruses, 18 betaherpesviruses, and 18 gammaherpesviruses from the family *Herpesviridae* (Figure 3). The ML phylogenetic tree classified the three identified suid herpesviruses into distinct subfamilies with robust bootstrap support (≥87%). PRVs clustered within the *Alphaherpesvirinae* subfamily, forming a monophyletic clade with reference PRV strains and other mammalian alphaherpesviruses. PoCMV was grouped within *Betaherpesvirinae,* aligning with porcine and primate betaherpesvirus lineages, while PLHV fell into *Gammaherpesvirinae*, clustering with ungulate gammaherpesviruses and the reference PLHV strain. The tree topology confirms that the three viruses are phylogenetically distinct and consistent with their established taxonomic classifications.

### 3.3. Genetic Diversity of PoCMV

**Figure 4:**
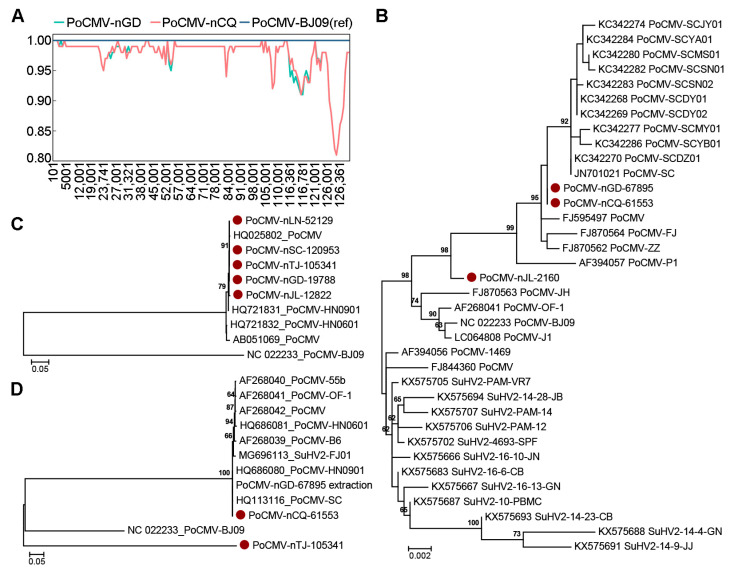
Genetic variation and maximum likelihood (ML) phylogenetic tree of PoCMV. (**A**) Whole-genome similarity analysis of two PoCMVs identified in the present study and the reference strain BJ09. The ML trees based on the full-length sequences of the PoCMV *UL39* (*gB*) gene (**B**), *UL57* (*MCP*) gene (**C**), and *UL38* (*DPOL*) gene (**D**) were constructed using MEGA 6.06 with 1000 bootstrap replicates. PoCMVs identified in this study are labelled by red solid circles.

PoCMV was the most widely distributed species of SuHVs in this study. To further understand its genetic variation, we first compared the full-length genome nucleotide sequences of the newly identified strains (nGD and nCQ) with the only publicly available PoCMV genome (strain BJ09). The two novel PoCMVs shared more than 99% nucleotide identity with each other. When aligned against strain BJ09, most genomic regions exhibited approximately 95% sequence similarity. Notably, a sharp decrease in identity (down to 80%) was observed in the 3′terminal region of the genome, indicating substantial genomic variation among Chinese PoCMV strains (Figure 4A).

Given the evident whole-genome genetic differences, we further selected three representative functional genes, namely, UL39 (encoding glycoprotein B, *gB*), UL57 (encoding major capsid protein, *MCP*), and UL38 (encoding DNA polymerase, *DPOL*), to construct ML phylogenetic trees for an in-depth evolutionary analysis of PoCMV prevalence in China. These genes were chosen due to their high conservation and conventional use in phylogenetic analyses of herpesviruses: *gB* is a classic taxonomic marker for viral lineage classification; *MCP* exhibits strong sequence conservation among circulating strains; and DNA polymerase provides reliable resolution for long-term evolutionary divergence. In the *gB* phylogenetic tree, two newly identified strains clustered in a sub-branch together with PoCMV strains reported in Sichuan Province, whereas the third novel strain formed an independent branch, indicating a distinct evolutionary trajectory (Figure 4B). In the *MCP* tree, the newly detected strains exhibited an extremely close evolutionary relationship with all Chinese isolates except for the BJ strain, which formed a separate branch, indicating that the *MCP* gene of Chinese PoCMVs is highly conserved (Figure 4C). In the *DPOL* tree, two new strains (PoCMV-nGD-67895 and PoCMV-nCQ-61553) showed a close evolutionary relationship with multiple reference PoCMV strains from China (e.g., AF268040_PoCMV-55b, AF268042_PoCMV-HN0601, and HQ113116_PoCMV-SC). Notably, the strain PoCMV-nTJ-105341 formed a distinct long branch, indicating significant genetic divergence from other PoCMV strains (Figure 4D). Together, PoCMV strains were divided into at least two major evolutionary clades, with new isolates distributed across different branches, suggesting the presence of genetically diverse circulating variants in Chinese pig populations.

### 3.4. Genetic Diversity of PRV

**Figure 5:**
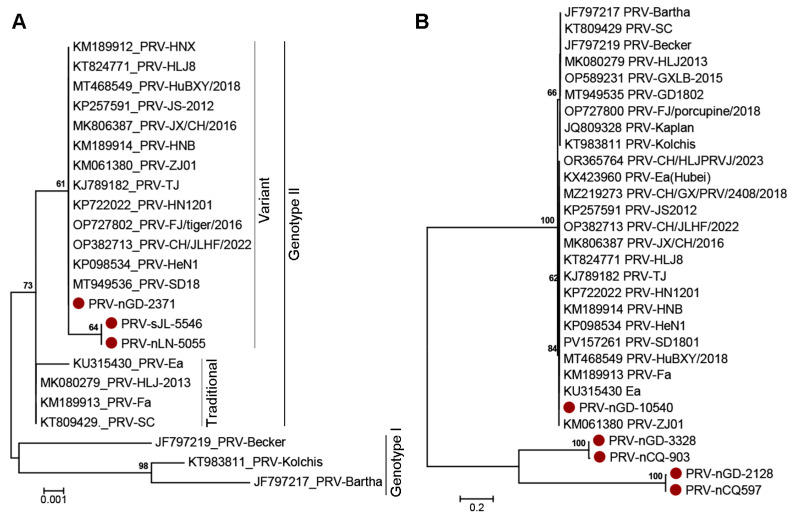
Maximum likelihood (ML) phylogenetic trees of PRV. The ML trees were constructed using MEGA 6.06 with 1000 bootstrap replicates based on the partial *gB* gene fragment (positions 17,497–18,096) (**A**) and the UL37 gene fragment (position 45,226–45,862) (**B**) of PRV. PRV strains identified in this study are labelled by red solid circles.

The evolution and genetic diversity of PRV in China have been extensively investigated. Previous studies revealed that PRV strains predominantly belong to genotype II and have diverged into two clades: classical and variant groups [25]. To assess the evolutionary characteristics of the newly identified PRV strains, ML phylogenetic trees were constructed based on partial sequences of the *gB* and UL37 genes. In the *gB*-based tree, all newly detected PRVs clustered within the variant genotype II clade (Figure 5A). The UL37 phylogenetic tree similarly revealed clear differentiation between classical and variant PRV lineages. Although all novel strains grouped within the variant genotype subclade, PRV-nGD-3328, PRV-nCQ-903, PRV-nGD-2128, and PRV-nCQ-597 formed two pairs of well-supported sub-branches, each with a bootstrap value of 100, suggesting that they may have originated from an ancestor distinct from previously reported viruses (Figure 5B). Phylogenetic analyses of both the *gB* and *UL37* genes consistently indicate that the PRVs identified in this study belong to the currently dominant variant genotype II and form a closely related evolutionary cluster. These results are consistent with the contemporary epidemiological pattern of PRV in China, where variant strains have replaced classical strains as the predominant circulating lineage.

### 3.5. Genetic Diversity of PLHV

**Figure 6:**
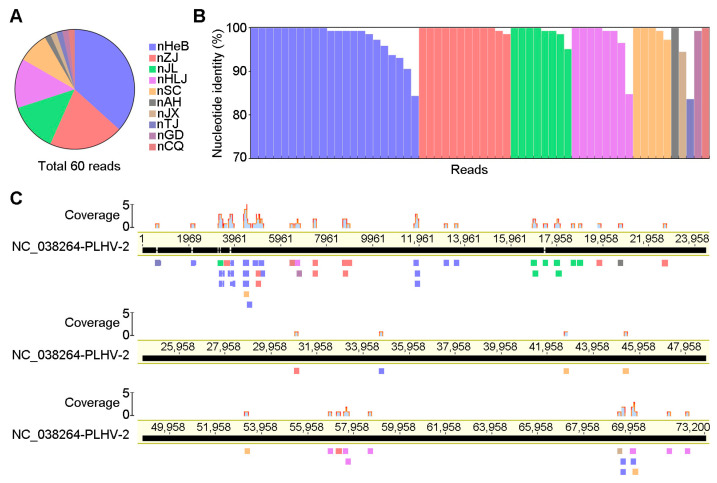
Genetic diversity analysis of PLHV. (**A**) Distribution of PLHV-related reads across sample libraries. (**B**) Nucleotide identity of reads annotated to the PLHV reference genome. (**C**) Schematic diagram showing the genomic distribution of mapped reads along the PLHV reference genome (NC_038264).

PLHV, an emerging oncogenic pathogen implicated in post-transplant lymphoproliferative disorder (PTLD) in xenotransplantation models, represents a significant zoonotic threat and holds pivotal etiological importance in veterinary virology. Owing to its ultra-low viral load in our study, we recovered only a single contig corresponding to PLHV, which exhibited a nucleotide identity of 70.8% to the reference strain (NC_038264). De novo assembly and read mapping analysis against the PLHV reference genome retrieved a total of 60 valid reads from 10 distinct libraries (Figure 6A). These reads demonstrated a high level of sequence similarity, with identities ranging from 83.67% to 100% (Figure 6B). Genomic distribution analysis revealed that these reads were scattered across disparate regions of the PLHV genome (Figure 6C). Specifically, while substantial reads mapped to conserved coding loci, including the glycoprotein B (*gB*) and *gp45* genes, the presence of large sequence gaps between contiguous reads hindered the generation of full-length consensus sequences. Taken together, our results support the circulation of potential PLHV variants in Chinese pig populations, revealing substantial sequence homology between these new strains and previously described PLHV isolates.

## 4. Discussion

Herpesviruses exhibit strong host specificity and the ability to establish latent infections. They can infect a wide range of vertebrates, posing substantial threats to both public health and animal husbandry [2,26,27,28]. Importantly, SuHVs are widely recognized for causing considerable economic losses in the global swine industry. In this study, we conducted a large-scale metaviromic analysis on Chinese SuHVs, revealing the detection patterns and genetic diversity of PRV, PoCMV, and PLHV in pig populations. Our findings provide a comprehensive overview of the current circulation profile of SuHVs in China and offer valuable insights for developing targeted prevention and control strategies against these viruses.

PRV has been the most extensively studied SuHV in pigs due to its high pathogenic potential [3,29,30]. However, the other two SuHVs have rarely been targeted in previous epidemiological investigations, resulting in a lack of data to evaluate their potential impact on the swine industry [7,12,31,32]. The present study focused on the large-scale detection of all three SuHVs using metaviromic analysis and revealed a strikingly high abundance and considerable genetic diversity of PoCMV in Chinese pig populations. This finding warrants future epidemiological studies to assess the impact of this virus on swine health and production. Importantly, we found that viral loads in nasal swabs were significantly higher than those in serum samples, which aligns with previous reports indicating that this virus primarily targets the nasal mucosa and is shed via the nasal cavity [12,33]. This indicates that nasal swabs represent the optimal specimen type for monitoring the circulation of this virus in pig populations. We obtained the full-length genomic sequences of two PoCMV strains, which shared high nucleotide identity with the PoCMV reference strain BJ09, indicating that Chinese PoCMV isolates are closely genetically related. Nevertheless, genetic variations were detected in several genomic regions. Further phylogenetic analyses based on the *gB*, *MCP*, and *DPOL* genes revealed the existence of at least two genetic subclades circulating across different provinces of China. This finding expands upon previous reports, which only indicated the genetic conservation of PoCMV strains identified in Sichuan Province [31].

Although present at low abundance, PLHVs were identified in ten sample pools, confirming that this virus is indeed circulating within Chinese pig populations. Notably, in the present study, PLHVs were exclusively detected in nasal swabs. Previous studies have demonstrated that PLHVs are well-characterized gammaherpesviruses with a strong tropism for lymphoid tissues, including lymph nodes and the spleen, where they establish persistent latent infections. Correspondingly, viral loads in peripheral tissues such as the nasal mucosa are typically very low, even in naturally infected animals [8,34,35]. This distinct tissue tropism also explains why only a very small number of valid reads (*n* = 60) and a single assembled contig were obtained throughout the present study, which is largely attributable to the fact that the primary predilection tissues of PLHV were not included in our sampling scheme.

The single assembled PLHV contig shared high nucleotide identity with the PLHV reference strain PLHV-2 (NC_038264), indicating a close genetic relationship with existing documented isolates. However, the limited and fragmented genomic data hinder detailed and robust inferences regarding its genetic variation and evolutionary trajectory. To date, large-scale epidemiological investigations and comprehensive genomic analyses of PLHV remain scarce worldwide, leaving significant gaps in understanding its epidemiological circulation, genetic diversity, and potential pathogenic effects on swine health [34,35]. To address these limitations, future surveillance should prioritize sampling of known target organs, including lymph nodes and whole blood, in addition to nasal swabs. When combined with viral enrichment strategies and full-genome sequencing, this approach will enable a more accurate assessment of PLHV circulation, genetic diversity, and epidemiological characteristics in domestic pig populations.

Despite conducting a large-scale virion-enriched metagenomic analysis in this study, several limitations may have introduced potential biases affecting the findings and conclusions. Approximately 9700 individual samples were pooled into 22 sequencing libraries, which greatly facilitated downstream analyses and reduced experimental costs; however, this approach likely reduced the detection sensitivity for detecting low-abundance viruses. Consequently, the present findings only reflect viral detection patterns at the pooled library level and may deviate from the actual viral prevalence in individual pigs. Additionally, samples were only collected from roughly half of the provinces in China, meaning the results may be biased and insufficient to reflect a comprehensive, nationwide distribution profile of SuHVs. Comparisons of viral detection rates and abundance across different sample types were also potentially affected by unequal sample sizes among the sampled provinces. Finally, the phylogenetic trees constructed in this study were based on partial sequences of target genes, which may yield slight discrepancies in topological structure compared with phylogenetic analyses performed using full-length gene sequences.

## 5. Conclusions

In conclusion, this large-scale, virion-enriched, metagenomic study systematically profiled the detection rates, geographic distribution, and genetic diversity of three SuHVs (PRV, PoCMV, and PLHV) in Chinese pig populations. PRV exhibited a restricted high-abundance distribution, concentrated in Jilin, Guangdong, and Heilongjiang provinces. PoCMV was ubiquitous with stable high abundance across all sampled regions and displayed considerable genetic diversity, with two distinct genetic subclades. PLHV was sporadically detected at low levels, exclusively in nasal swabs, consistent with its natural tropism for lymphoid tissues and low viral loads in peripheral tissues. Nasal swabs were confirmed as the optimal specimen for SuHV surveillance. We also obtained two full-length PoCMV genomes to enrich public genomic data. Despite some limitations, this study provides key insights for SuHV prevention and control and further relevant research.

## Data Availability

The complete sequences of the *gB*, *MCP*, and *DPOL* genes reported in this study have been submitted to the GenBase of the National Genomics Data Center with accession numbers C_AA167569.1 to C_AA167578.1 and can be accessed publicly at https://ngdc.cncb.ac.cn/genbase.
